# Role of shear-wave and strain elastography to differentiate malignant vs benign subpleural lung lesions

**DOI:** 10.1097/MD.0000000000024123

**Published:** 2021-01-08

**Authors:** Andrea Boccatonda, Valentina Susca, Gian Luca Primomo, Giulio Cocco, Sebastiano Cinalli, Velia Di Resta, Laura Martino, Felice Mucilli, Stefano Marinari, Francesco Cipollone, Cosima Schiavone

**Affiliations:** a Unit of Ultrasound in Internal Medicine, Department of Medicine and Science of Aging, “G. d’Annunzio” University; b Pneumology Department, SS Annunziata Hospital, Chieti; c Division of pathology, ASST della Valtellina e dell’Alto Lario, Sondrio; d Department of General and Thoracic Surgery, University Hospital “SS. Annunziata”; e Department of Medicine and Aging, Center of Aging Science and Translational Medicine (CESI-Met), Via Luigi Polacchi, Chieti, Italy.

**Keywords:** cancer, elastosonography, lung, thorax, ultrasound

## Abstract

Elastosonography is a non-invasive diagnostic method to evaluate tissue stiffness. The aim of our study was to demonstrate the applicability and efficacy of elastosonography to differentiate benign vs malignant subpleural lung lesions compared to clinical, radiological and histological findings.

We performed both strain and shear wave velocity (SWV) elastosonography on subpleural lung lesions. Moreover, we elaborated a composite score called ”elasto index”.

Fourteen patients, 10 males and 4 females were recruited. On strain elastography, 9 lesions showed a hard pattern (type 3), 3 lesions showed an intermediate pattern (type 2), and 2 lesions a soft pattern (type 1). All lesions showed a mean SWV value of 4.46 ± 2.37 m/second. The mean SWV for malignant lesions (n = 6) was 5.92 ± 2.8 m/second. The mean SWV for benign lesions (n = 8) was 3.36 ± 1.20 m/second. SWV shows an area under the curve (AUC) of 0.792, and the Youden index shows a value of 3.6 m/second. The ROC curve elaborated for the diagnosis of malignancy by strain elastography showed an AUC of 0.688. ROC curve for the diagnosis of malignancy by elasto index demonstrated an AUC of 0.802.

SWV values obtained by ARFI elastosonographic method are higher in malignant lung lesions (mean SWV: 5.92 m/second) than in benign ones (mean SWV: 3.36); a composite score (elasto index) is characterized by better statistical significance for the differentiation of the lesions.

## 1 Introduction

For years, the lung has always been considered an organ that cannot be examined by ultrasound due to its air content (>90%), given that the air blocks the ultrasound signal due to the high difference in acoustic impedance between the superficial tissues of the chest. Indeed, the study of the artifacts deriving from the eco-structural differences between the various components of the rib cage becomes the keystone for the interpretation of multiple differential pathological findings. In recent years, the application of lung ultrasound has extended from the field of urgency to that of routine/ordinary study of patients with chronic lung diseases.^[1–3]^ Evaluation and follow-up of lung lesions are most difficult areas in the diagnostic field. Early diagnosis of lung cancer by radiological screening methods contributes to improving survival rates. Currently, low-dose computed tomography (CT), positron emission tomography (PET), and magnetic resonance (MR) are more commonly employed screening methods. Lung ultrasound is a method displaying several advantages, such as low-cost, real-time imaging and the absence of radiation. Elastosonography has been employed to determine tissue stiffness/elasticity, especially in the field of hepatology. Currently, thyroid, breast, and prostate are potentially investigable organs for non-invasive diagnostic imaging of nodules suspected for malignancy.^[4,5]^ While B-mode ultrasound may reveal morphological properties of tissues, elastosonography can provide quantitative and qualitative information on tissue stiffness.^[4,5]^

Elastosonography is based on the emission of short-lived (0.03–0.4 ms) high-energy acoustic pulses generated by the probe, which induce a small amount of local displacement (1–20 μm) in the tissue. The so-called “shear waves” are generated following that displacement, which can be detected by the probe. Few published studies have experimented with the application of elastosonography in pulmonary diseases.^[6,7]^

The main aim of our study is to demonstrate the applicability and efficacy of elastosonographic measurements in differentiating benign vs malignant subpleural lung lesions (benign lesions such as pneumonia vs malignant cancer lesion) compared to clinical, radiological (CT/PET) and histological findings.

## 2 Material and methods

### 2.1 Study patients

We recruited patients displaying clinical, radiological and histological diagnosis of subpleural lung lesions, admitted on the Pneumology and Internal Medicine wards. Ultrasound examination were performed in Internal Medicine ultrasound unit. Patients with subpleural lung lesions of size <1 cm and those with pleural effusion were excluded. It was not possible to recruit a healthy control group due to the non-applicability of elastosonography to normally ventilated and non-pathological lung. After giving written and informed consent, 14 subjects (10 males), with a mean age of 67 (18–87) years, were enrolled in the study. The clinical characteristics of the study subjects are summarized in Table 1.

**Table 1:** Clinical findings of study patients.

Variable	Value
n	14
Age, years	67 (18–87)
Male sex, n (%)	10 (71.4)
Hypertension, n (%)	5 (35.7)
Atrial fibrillation, n (%)	1 (7.1)
Pulmonary hypertension, n (%)	2 (14.2)
Type 2 diabetes, n (%)	2 (14.2)
COPD, n (%)	4 (28.5)
IPF, n (%)	1 (7.1)
ACE-I, n (%)	0 (0)
ARB, n (%)	2 (14.2)
Diuretics, n (%)	3 (21.4)
Beta-blockers, n (%)	1 (7.1)
DOAC, n (%)	0 (0)
Warfarin, n (%)	1 (7.1)
PPI, n (%)	4 (28.5)

This work is the result of a retrospective analysis on data obtained during normal clinical practice from lung ultrasound scans performed between January 2018 and December 2019, in which elastosonography was used as a diagnostic completion, subject to the acquisition of informed consent from each patient. Therefore, no IRB approval is available for this work. From those preliminary observations, a prospective observational study protocol has been designed and is in the process of being approved by the relevant ethics committee.

### 2.2 Methods

The ultrasound examinations and elastosonography were performed by the same sonographer, who had at least 2 years of experience in the method. The ultrasound and elastosonographic examinations were performed with a convex probe, for the shear-wave method, and linear probe, for the strain method, with the EPIQ Philips device.

Shear waves are generated by ultrasound pulses of a given acoustic radiation strength. The velocity of the shear wave is then estimated by a Doppler effect on a region of interest (ROI) and is related to the stiffness or elasticity of the medium. This shear wave velocity can be used to calculate the tissue stiffness by the formula E = ρc2, where E is the elasticity of the tissue (Young modulus, kPa), ρ is the density of the tissue (kg/m^3^) etc. is the speed of the shear wave (m/second).

**Figure 1:**
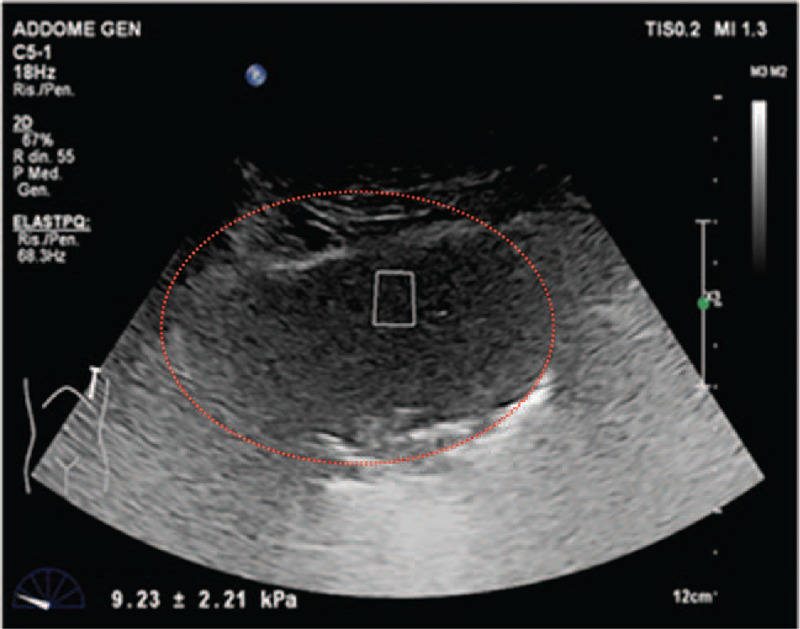
SWV quantification was performed on the axial plane obtained from central area of the lesion. The region of interest (ROI) was located on the solid and well visualized sections of the lesions, avoiding the necrotic and/or cystic areas. SWV has been automatically quantified in meters per second (m/second). The lung lesion is highlighted by the red dotted line.

During the B-mode ultrasound examination, patients were on supine position on normal breathing. After B-Mode ultrasound examination, the quantification of the shear wave velocity (SWV) was performed by placing the probe on intercostal scan, to avoid the mechanical effort given by the presence of the ribs. The transducer was placed in contact with the skin slightly to minimize operator-dependent pressure on the chest. An acoustic radiation force impulse (ARFI) technique was used combined with virtual tissue imaging software (Philips EPIQ). During SWV measurement, the patient was asked to hold his breath and not to cough, strain or move. SWV quantification was performed on the axial plane obtained from central area of the lesion. The region of interest (ROI) was located on the solid and well visualized sections of the lesions, avoiding the necrotic and/or cystic areas (Fig. 1). SWV within the ROI has been automatically quantified in meters per second (m/second). Measurements were repeated for 3 consecutive times. SWV means for each lesion were measured and then compared with clinical, radiological and histological data. Subsequently, we elaborated a subdivision into 4 groups based on the results of SWV analysis:

Group I: 1–3 m/second;
Group II: 3–6 m/second;
Group III: 6–9 m/second;
Group IV: >9 m/second.

**Figure 2:**
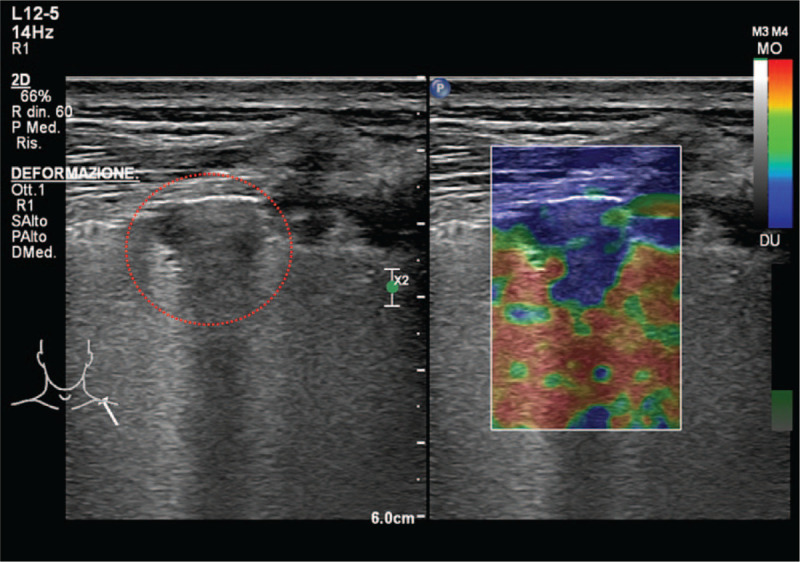
B-mode and strain elastosonography of sub-pleural lung consolidation; the lesion displayed a blue pattern >75% (hard lesion). The lung lesion is highlighted by the red dotted line.

In relation to strain technique, the color box was located in the solid and well-visualized sections of the lesions; the qualitative study, based on the color scale encoding the blue color as hard and red as soft, led to the classification of the lesions based on the homogeneity and percentage of presence of the red signal (Type I: blue color <25% - soft le-sion; Type II: blue color 25% to 75% - intermediate lesion; type III: blue color >75% hard lesion) (Fig. 2).

Eventually, in order to elaborate a score accounting both the SWV (quantitative) and the strain (qualitative) methods, we elaborated an “elasto index”: elasto strain value + SVW group value (Minimum value 2 - maximum value 7). This score was compared with the definitive histological diagnosis of the lesions.

## 3 Statistical analysis

Univariate associations were evaluated by the Spearman rank correlation test. Data are presented as median interquartile range (IQR). *P* values <.05 were considered statistically significant. Furthermore, ROC curves have been elaborated for the calculation of the AUC, of the sensitivity and specificity of the elastosonographic values. All tests were two-sided and analyzes were performed using the SPSS statistical package (see 16.0, APSS, Chicago, IL, USA).

## 4 Results

**Figure 3:**
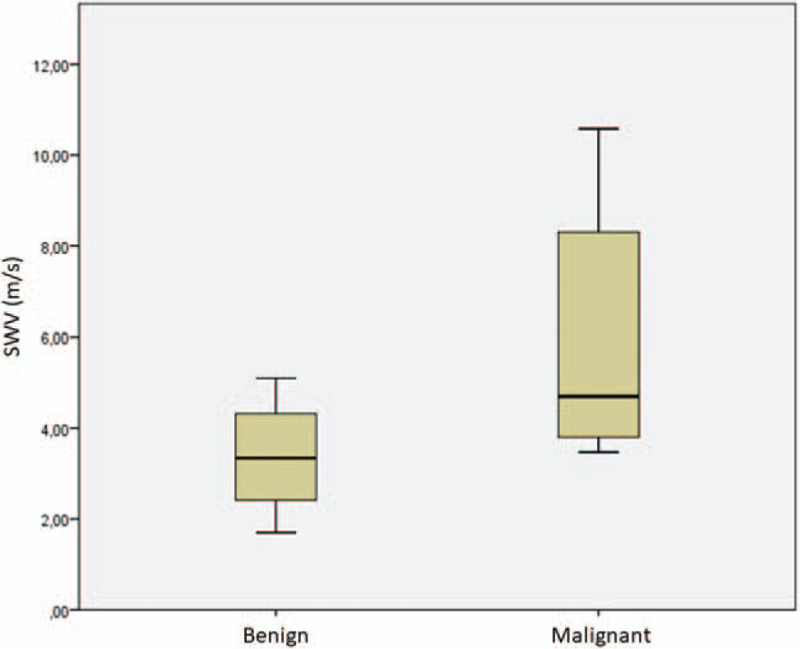
Median SWV values in comparison between patients with malignant and benign lesions. Patients with histological diagnosis of malignancy present higher values.

**Figure 4:**
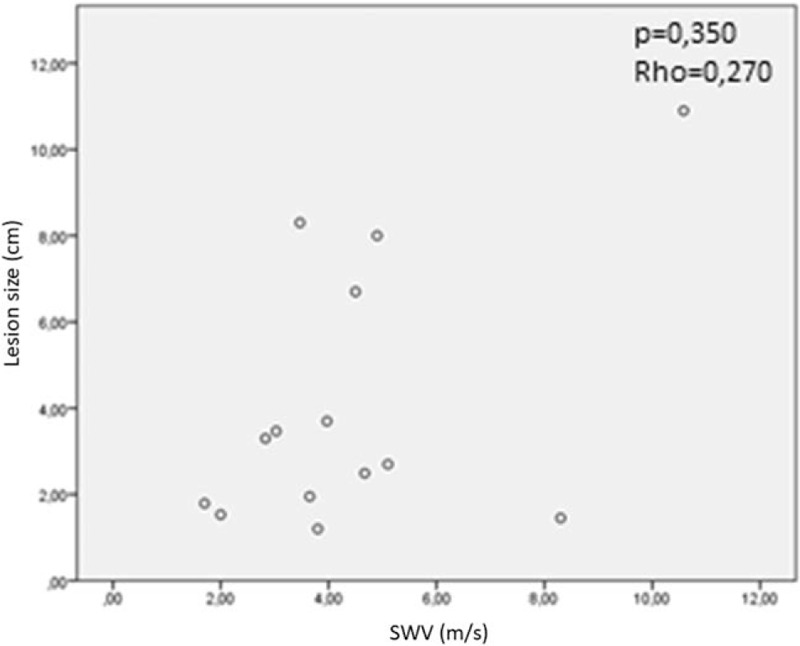
Correlation between SWV values and lesion sizes; SWV value is not dependent on the size of the lesion.

**Figure 5:**
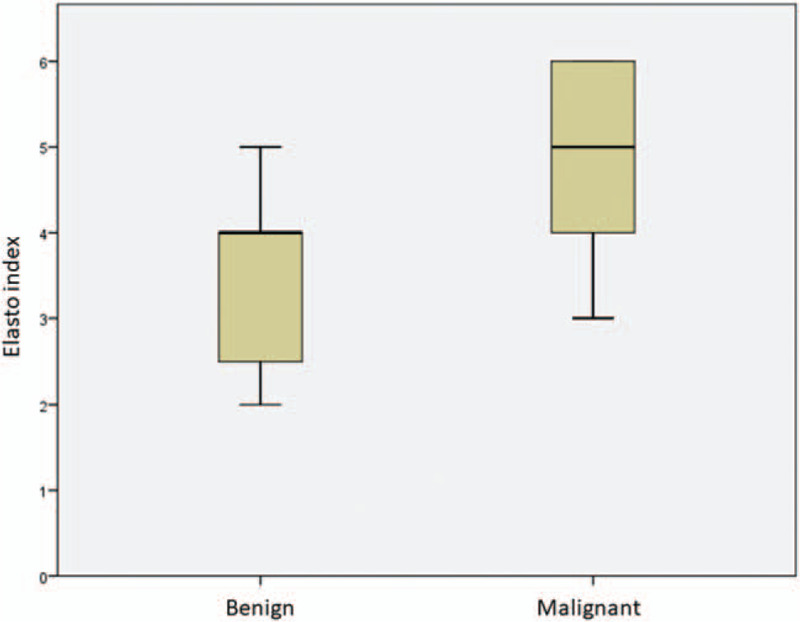
Comparison of elasto index (EI) values between subjects with malignant and benign lesions; patients with malignant lesions are characterized by higher EI values.

**Figure 6:**
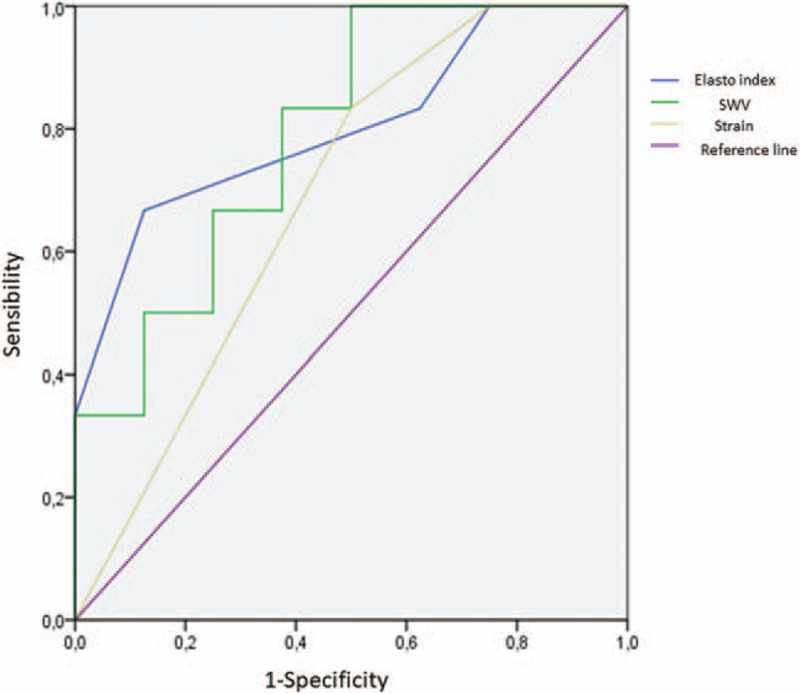
Comparison of ROC curves of the 3 methods examined for the diagnosis of lung malignancy.

Fourteen patients, 10 males and 4 females were recruited. The mean age was 67 years. The baseline characteristics of the patients are listed in Table 1. It was possible to detect the lesions reported to the radiological examinations (chest CT) in all examined cases (see Table 2). The mean size of the lesions was 4.1 cm. On strain elastography analysis, 9 lesions showed a hard pattern (type 3), 3 lesions showed an intermediate pattern (type 2), and 2 lesions a soft pattern (type 1). The elastosonography performed on all lesions showed a mean SWV value of 4.46 ± 2.37 m/second. The mean SWV value for malignant lesions (n = 6) was 5.92 ± 2.8 m/second. The mean SWV value for benign lesions (n = 8) was 3.36 ± 1.20 m/second (Fig. 3). On statistical analysis, SWV values did not significantly correlate with age (*P* = .259; Rho = −0.324) and lesion size (*P* = .350; Rho = 0.270) (Fig. 4). If a ROC curve is elaborated for the malignancy diagnosis, SWV shows an area under the curve (AUC) of 0.792, and the calculation of the Youden index shows a value of 3.6 m/second. Regarding the strain elastography analysis, it did not show statistically significant correlations with age (*P* = .628; Rho = 0.142), and size of lesions (*P* = .686; Rho = 0.119). The ROC curve elaborated for the diagnosis of malignancy by strain elastography showed an AUC of 0.688, and the calculation of the Youden index showed a value of 2.5. Elasto index did not correlate with patient age and size of lesions. ROC curve elaborated for the diagnosis of malignancy by elasto index demonstrated an AUC of 0.802, and the calculation of the Youden index showed a value of 4 (Figs. 5 and 6) (Table 3).

**Table 2:** Comparison between different features of the examined lung lesions.

SEX	Strain Pattern	Stiffness (kPa)	Malignancy CT Features	Histological Features	Color-Doppler Flow
M	3	10,5	L	Choriocarcinoma metastasis	+
F	1	3,4	UD	Small cell lung cancer	0
M	3	4,6	L	Adenocarcinoma	0
M	3	4,5	UD	Adenocarcinoma	+
M	2	3,9	U	Pneumonia	0
F	2	5,1	UD	Pneumonia	0
M	3	2,8	U	Micotic pneumonia	0
M	3	3,8	UD	Small cell lung cancer	0
F	1	1,7	U	Pneumonia	0
F	3	3,6	U	Ab ingestis pneumonia	0
M	1	2,0	U	Pneumonia	0
M	3	3,0	U	Pneumonia	0
M	3	4,9	L	Squamous cell carcinoma	0
M	3	8,3	L	Squamous cell carcinoma	+

**Table 3:** Statistical analysis comparion between ROC curves for the diagnosis of lung malignacy.

	Area	STD error	Asintotic sign.	IC 95% (low)	IC 95% (max)
SWV	0,792	0,122	0,071	0,553	1,00
STRAIN	0,688	0,144	0,245	0,405	0,97
Elasto index	0,802	0,127	0,061	0,554	1,00

## 5 Discussion

The first data to report is that all lung lesions reported on the chest CT as subpleural have been evidenced by B-mode ultrasound. For years, lung ultrasound was not considered “feasible” due to the physical properties of the lung tissue due to high difference in acoustic impedance between the tissues. Moreover, the main limitation of lung ultrasound is that the consolidation should involve the peripheral portion of the lung and “touch” the pleural line, thus changing the physiological A line pattern created by the normal air/chest wall tissues interface. Furthermore, although we can estimate the lesion size on ultrasound, it almost never corresponds to the real 1, as the margins are often blurred and the limit in depth is almost never clear.

By B-mode ultrasound, lung atelectasis, inflammatory consolidations, tumor masses, pulmonary infarcts all appear with a substantially gray scale echostructure, as like to the sonographic feature of the liver, from which the term “hepatization” of lung tissue. Indeed, several studies and authors have attempted to create a differential semiotics of the various lesions, based on the presence of signs such as shape, margins, echostructure, evidence of vascularization, the presence of air and fluid bronchograms.^[8]^ However, no specific sign has been shown to be able to differentiate malignant lesions from benign ones with absolute specificity. Particularly, air bronchograms are present both in tumor lesions and in pneumonia consolidations. Furthermore, the color-Doppler examination is possible in some lesions only, and it is not suggestive for the nature of the lesion (in our sample 3 lesions showed a clear color-Doppler flow).

Elastosonography is a new diagnostic tool employed to evaluate the stiffness or elasticity of a tissue. It is based on the physical concept that when external forces are applied to any tissue, a deformation of the tissue occurs. Elastosonography can measure this deformation, by processing a speed wave, and converting the data into ultrasound images, while the traditional B-mode ultrasound demonstrates the morphology of the tissue.

In our work, the usefulness of elastosonography to differentiate subpleural lung lesions/masses was evaluated and SWVs were measured by ARFI technique. The stiffness of malignant tumor masses was higher (mean SWV value of 5.92 ± 2.8 m/second) than benign ones (mean SWV 3.36 ± 1.20 m/second). In particular, the SWV value of 3.6 m/second demonstrates the best diagnostic accuracy.

We also employed the strain elastography to evaluate different patterns, such as those defined as “hard” with a high percentage of rigid tissue, to those “soft” with a low percentage of rigid tissue.

By combining the information given by the 2 methods into a single score, we developed an index, which showed the best predictive performance on statistical analysis. Elasto index presented an area under the curve of 0.802, and from the calculation of the Youden index, the value showing the best characteristics in terms of sensitivity and specificity is that of 4.

Our data seem to agree with previous works showing higher stiffness values for malignant lung lesions. In the work performed by Ozgokce et al, the mean of SWV values was 3.50 ± 0.69 m/second for malignant lesions. Furthermore, authors reported that when the SWV cut-off value was set at 2.47 m/second, the sensitivity and specificity were 97.7%.^[9]^ In another study by Sperandeo et al, a significant increased rigidity of squamous cell lung tumors (4.67 ± 0.492) was observed compared to other types of lung cancer (*P* < .005) and compared to pneumonia (2, 35 ± 0.48).^[6]^

**Figure 7:**
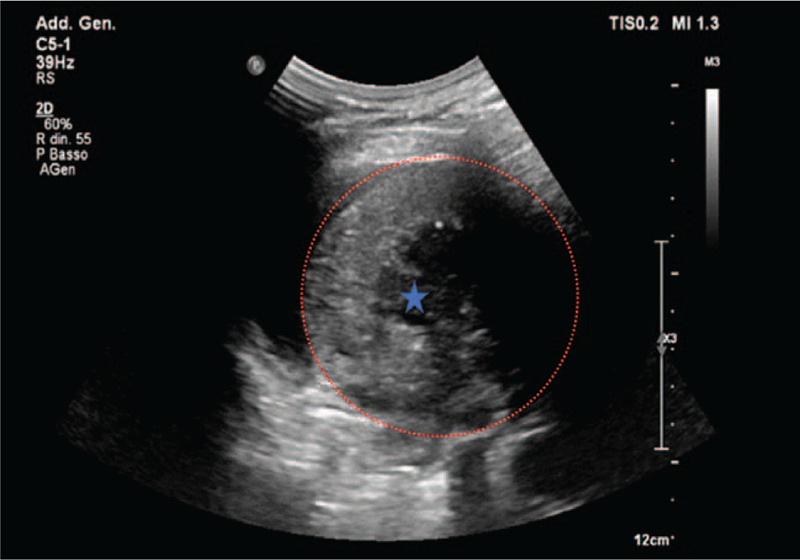
B-mode imaging representing lung subpleural mass with a necrotic core (anechoic area); in particular, it is a massive metastasis from ovarian choriocarcinoma. SWV value was of 10.58 m/second. the lung lesion is highlighted by the red dotted line; blue star highlights the necrotic core.

Similar to our data, in the study by Sperandeo et al patients age and the size of the masses (3.06 ± 0.88 cm) were not significantly associated with histopathological types.^[6]^ A further study by Lim et al found that the velocity rate of deformation of primary lung tumors was higher than metastatic lesions.^[10]^ In contrast, Ozgokce and collegues demonstrated that the mean SWV (4.12 m/second) of metastatic lesions was higher than primary lung cancers (3.43 m/second).^[9]^ In our case, the highest SWV value was obtained on a metastatic choriocarcinoma lesion, with values of 10.58 m/second (Fig. 7).

We also believe that the SW method can be useful in determining the area to be biopsied in the widest subpleural lung lesions, with heterogeneous and necrotic areas. In our study 2 lesions clearly showed a necrotic core. Therefore, the accuracy in selecting the “target” point in biopsy procedures could be increased, thus improving the safety profile of the procedure.^[6,11]^

In our opinion, current data on elastosonography to study subpleural lung lesions are almost “primordial”. Indeed, the number of patients examined is small in different studies, and the methods of study are often not comparable; elastosonographic methods (ARFI vs TE), the probes and, finally, dedicated machines and softwares are different. Moreover, in some cases the final diagnosis of the nature of the lesion was not supported by histological data. The main limitation of the method seems to be due to the peculiarity of the lung tissue; a large proportion of non-peripheral lesions cannot be examined by ultrasound; secondly, it is not possible to perform an ultrasound study of lesion margins, although some data showed an excellent specificity in the study of parietal infiltration of lesions.

Given the lack of uniformity, there is currently no threshold value or classification, as for the Metavir scale for hepatic stiffness in hepatitis, which can classify and discriminate the nature of the lesions. We proposed both a scale of values regarding the evaluation of SWV and a strain classification to differentiate lesions; finally we elaborated an elasto index in order to merge the qualitative information of the strain and the quantitative information by the SWV, so to increase the diagnostic accuracy.

## 6 Limitations of the study

There are some limitations in our work. First, since we used a linear probe in some patients, the subcutaneous adipose tissue in some subjects may hinder the correct measurement of elastosonographic methods. The second limitation was that all patients had a subpleural CT lesion; it was not possible to examine the consolidations that did not reach the pleural line; furthermore, due to the peculiarity of the application of the method to the lung tissue, it was not possible to examine a control group or assess marginal differences between the stiffness of the pathological tissue and that normally ventilated. A further limitation is that our study included a limited number of cases.

## 7 Conclusions

Our study demonstrates that SWV values obtained by ARFI elastosonographic method are higher in malignant lung lesions than in benign ones; when the numerical data of the SWV is combined with the qualitative data of the strain elastography thus obtaining a composite score, a good statistical significance is reached for the differentiation of lung lesions. Therefore, the present study may reinforce the preliminary data on the application of transthoracic elastosonography in evaluating subpleural lung lesions. Transthoracic elastosonography can be a non-invasive, repeatable method, without contraindications, executable even at bed-side, to evaluate subpleural lesions. In the future, multicenter studies will be required, performed on a large sample of subjects, with uniformity of methods, which may lead to more definitive conclusions regarding the application of elastosonography to the lung field, and the possible creation of a scale or cut-off values.

## Acknowledgments

Thanks to Noemi Dell’Orso for her assistance.

## Author contributions

**Conceptualization:** Andrea Boccatonda, Valentina Susca.

**Data curation:** Andrea Boccatonda, Valentina Susca, Gian Luca Primomo, Sebastiano Cinalli, Velia Di Resta, Laura Martino, Stefano Marinari.

**Funding acquisition:** Cosima Schiavone.

**Investigation:** Gian Luca Primomo, Giulio Cocco.

**Resources:** Cosima Schiavone.

**Supervision:** Giulio Cocco, Felice Mucilli, Stefano Marinari, Francesco Cipollone, Cosima Schiavone.

**Validation:** Felice Mucilli, Stefano Marinari, Francesco Cipollone, Cosima Schiavone.

**Writing – original draft:** Andrea Boccatonda.

**Writing – review & editing:** Andrea Boccatonda, Cosima Schiavone.
